# Autosomal Dominant Cutis Laxa in an Adolescent Male: A Rare Clinical Entity

**DOI:** 10.7759/cureus.106483

**Published:** 2026-04-05

**Authors:** Mahwish Hassan, Sulhera Khan, Zara Saeed, Sonia Golani, Humaira Talat

**Affiliations:** 1 Dermatology, Dow University of Health Sciences, Civil Hospital Karachi, Karachi, PAK; 2 Internal Medicine, Jinnah Postgraduate Medical Centre, Karachi, PAK

**Keywords:** autosomal dominant cutis laxa, connective tissue disorder, elastolysis, eln gene, psychosocial impact

## Abstract

Cutis laxa is a rare connective tissue disorder characterized by defective elastic fiber formation, leading to loose, inelastic skin and a prematurely aged appearance. Autosomal dominant cutis laxa (ADCL) is an uncommon inherited form that generally presents later in life with relatively mild systemic involvement and is most commonly associated with mutations in the *ELN *gene. We report a 17-year-old boy with a five-year history of progressive generalized skin laxity that began at 12 years of age. The laxity initially involved the face and gradually extended to the neck and trunk. His medical history was notable for two prior inguinal hernia repairs. There was no family history of similar complaints, and joint hyperextensibility was absent. Histopathological examination revealed reduced and fragmented dermal elastic fibers, confirmed by Elastic Van Gieson staining. Cardiovascular, pulmonary, and abdominal evaluations were unremarkable. The patient developed major depressive disorder secondary to cosmetic disfigurement. This case highlights the clinical variability of ADCL in adolescence and the importance of multidisciplinary management, psychosocial support, and long-term follow-up.

## Introduction

Cutis laxa, also known as elastolysis, is a rare connective tissue disorder characterized by abnormalities in elastic fiber formation within the dermis, resulting in loose, inelastic skin and prominent folds that impart a prematurely aged appearance [1,2]. In addition to cutaneous laxity, the disorder may be associated with varying degrees of systemic involvement, affecting the pulmonary, cardiovascular, and gastrointestinal systems. Cutis laxa may be inherited or acquired, with inherited forms following autosomal dominant, autosomal recessive, or X-linked recessive patterns. Acquired cutis laxa can occur secondary to infections, autoimmune conditions, inflammatory dermatoses, or malignancies [3,4]. We report the case of a 17-year-old boy presenting to the dermatology outpatient department with progressive skin laxity beginning at the age of 12. Clinical findings, supported by histopathological examination, were consistent with a diagnosis of autosomal dominant cutis laxa (ADCL).

## Case presentation

A 17-year-old boy, previously healthy, presented with a five-year history of progressive, generalized skin laxity. He reported being in his usual state of health until the age of 12, when he first noticed looseness of the facial skin, which gradually extended to the neck and trunk over approximately 18 months. His past surgical history was notable for two inguinal hernia repairs conducted seven and five years before the current presentation.

There was no history suggestive of connective tissue fragility, including easy bruising or joint hyperextensibility. No family members had similar complaints. He was born to consanguineous parents via an uncomplicated term vaginal delivery and had achieved normal developmental milestones. The psychosocial impact of his condition had led him to leave school, and he expressed significant distress about his appearance.

**Figure 1:**
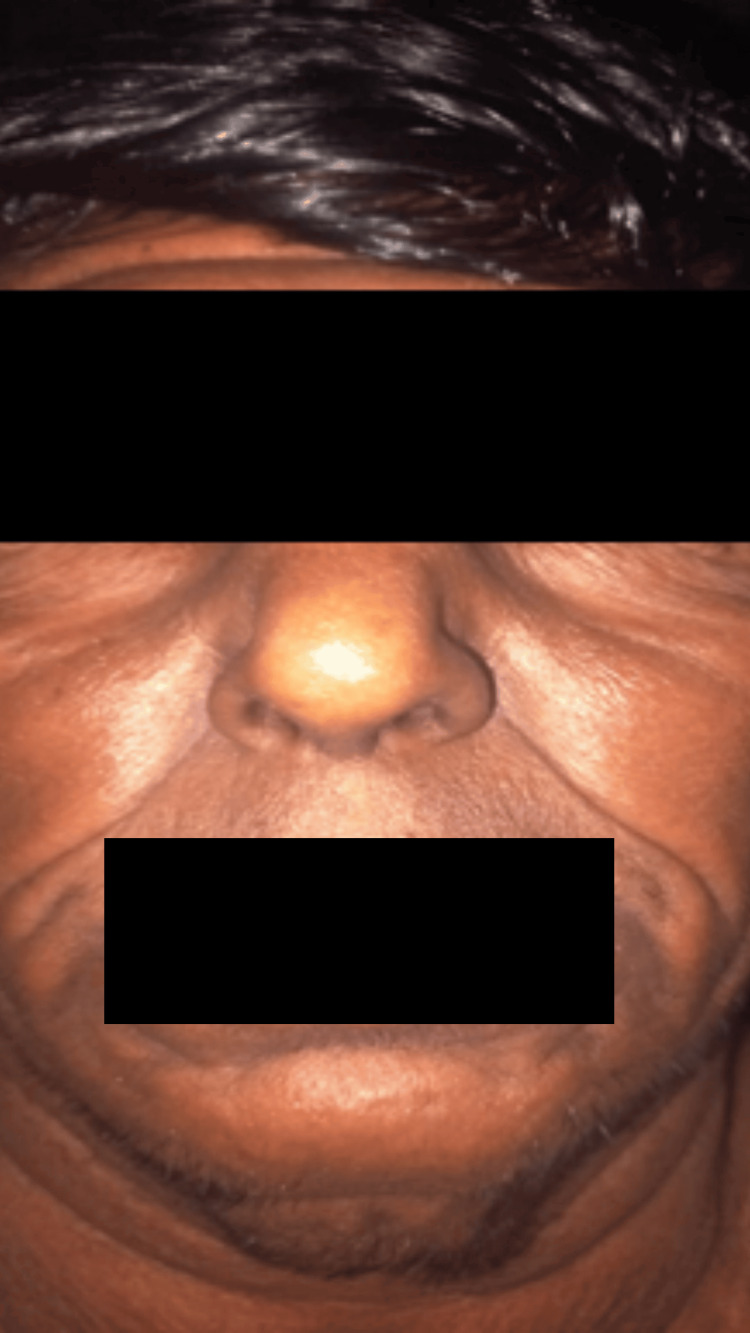
"Droopy" facial appearance with downward-slanting palpebral fissures, a broad and flattened nasal bridge, and sagging cheeks

**Figure 2:**
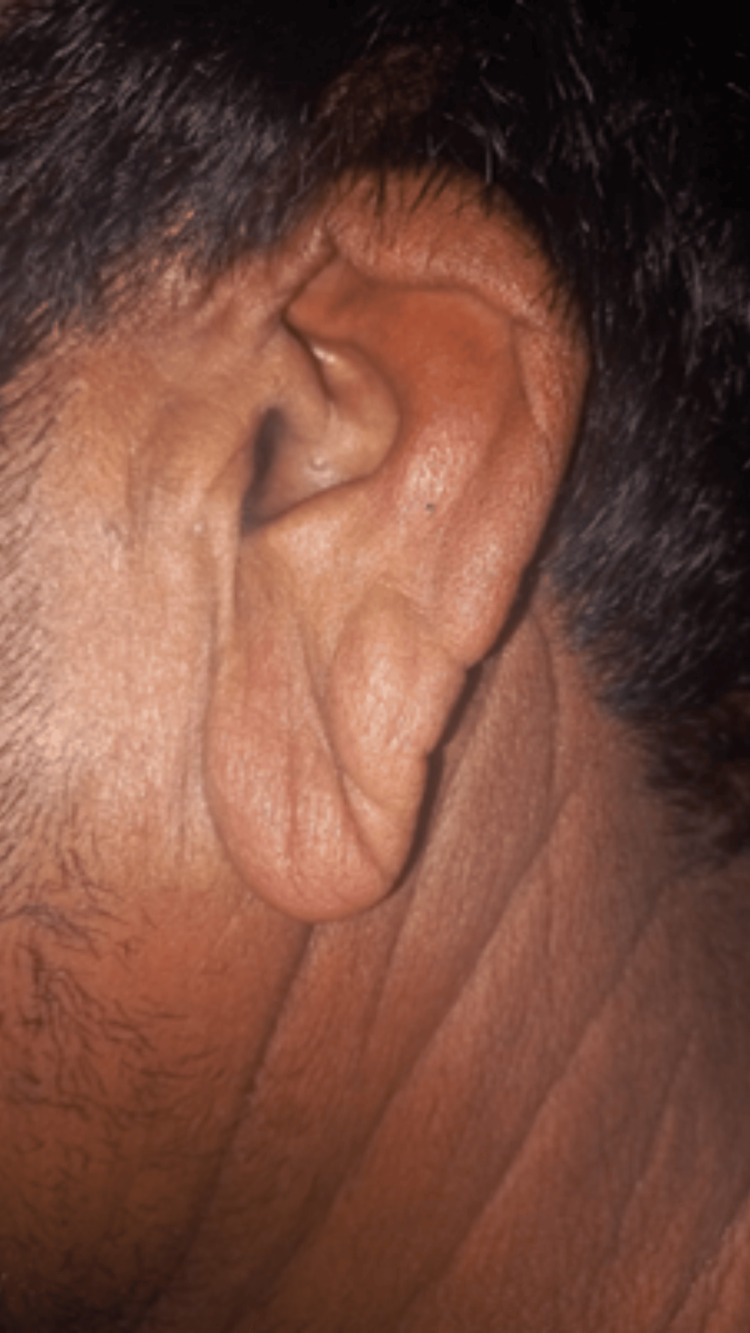
Pendulous, prominent earlobes

**Figure 3:**
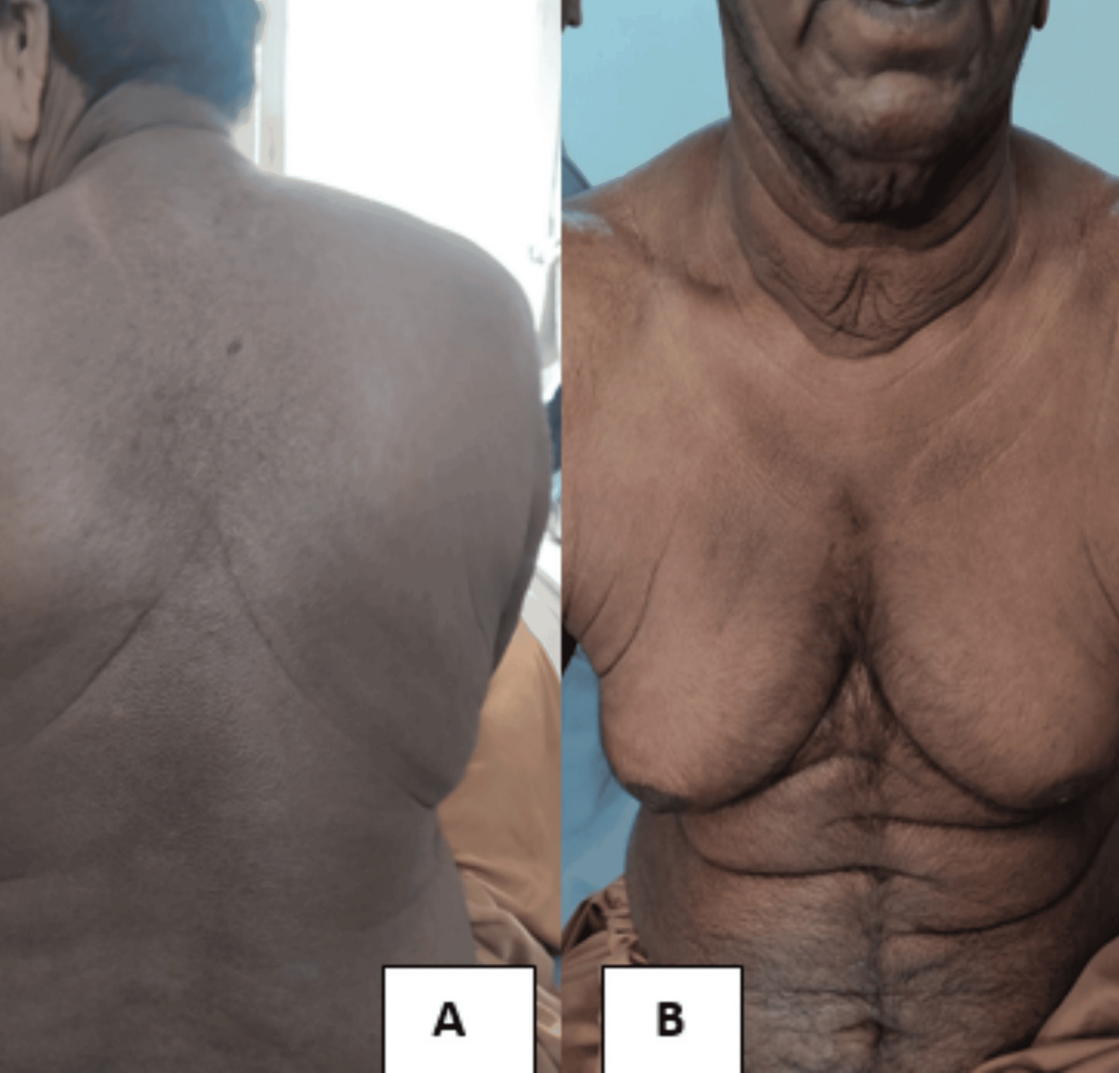
(A) Redundant skin folds visible over the back. (B) Prominent skin sagging over the abdomen

Cutaneous examination revealed generalized skin laxity involving the trunk and extremities, with the most striking changes on the face. He displayed a characteristic "droopy" facial appearance with downward-slanting palpebral fissures, a broad and flattened nasal bridge, sagging cheeks (Figure 1), and pendulous, prominent ear lobes (Figure 2). Redundant skin folds were visible over the abdomen (Figure 3A), with prominent skin sagging over the abdomen (Figure 3B), though the skin lacked hyperextensibility. Joint mobility was within normal limits.

A psychiatric assessment confirmed that he met Diagnostic and Statistical Manual of Mental Disorders (DSM-5) criteria for major depressive disorder [5].

**Figure 4:**
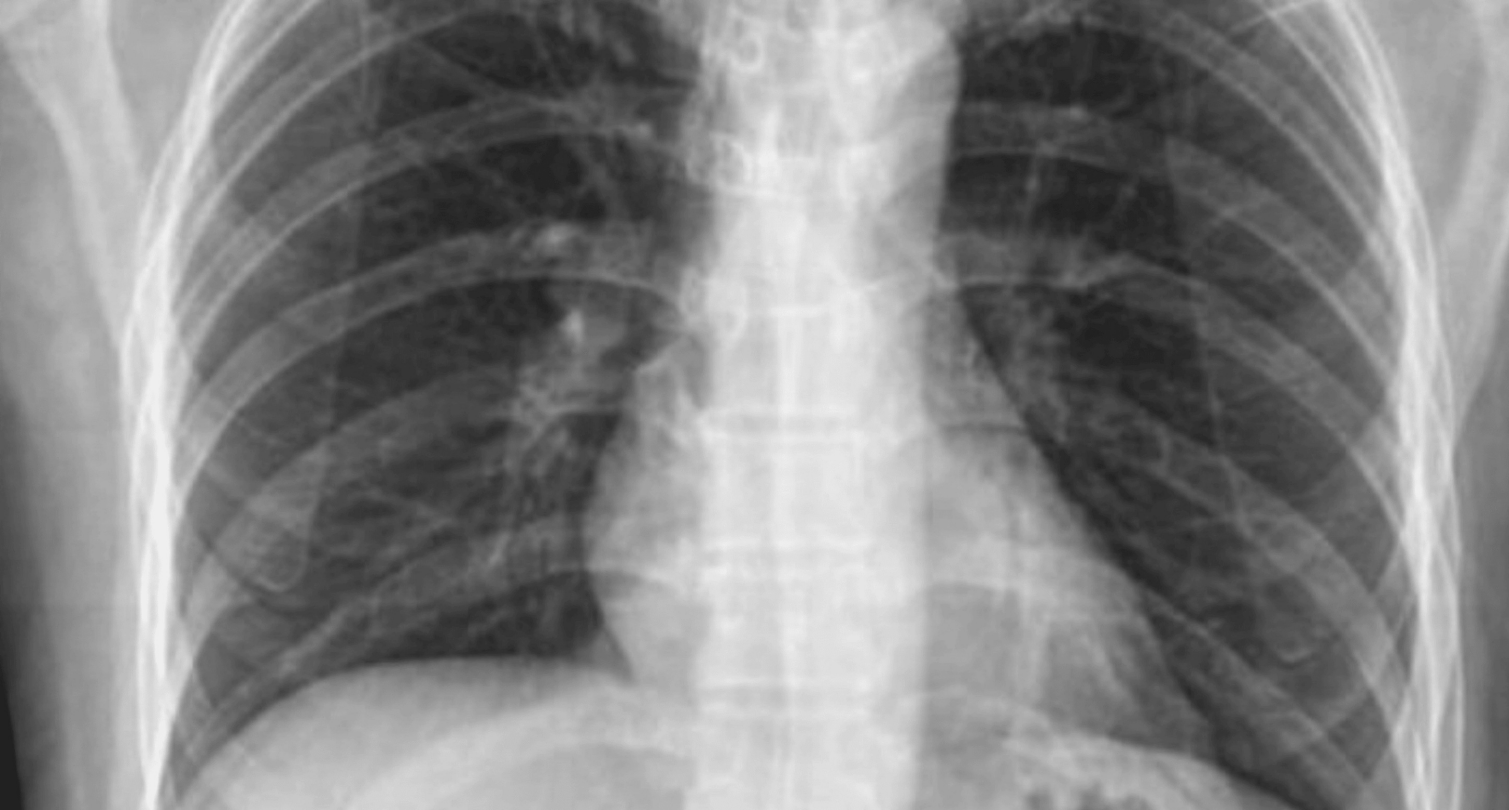
Normal chest X-ray (PA view)

Systemic evaluation was unremarkable. Routine laboratory tests, including complete blood count, renal and liver function tests, and autoimmune markers, were within normal ranges. Echocardiography to assess for cardiovascular involvement, chest radiography (Figure 4), and pulmonary function tests were carried out to exclude lung involvement, and abdominal ultrasonography revealed no abnormalities.

**Figure 5:**
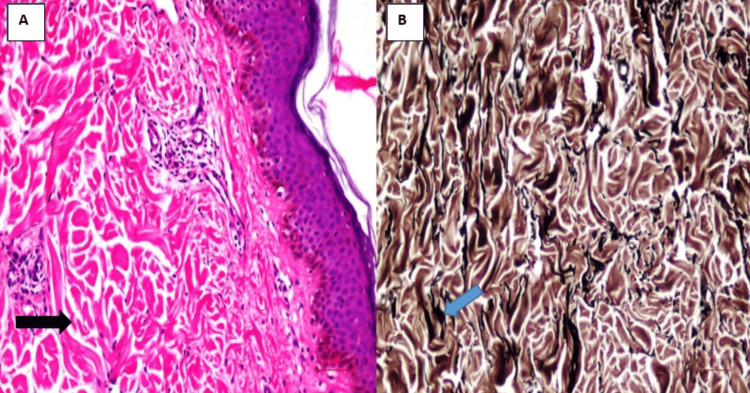
(A) Skin biopsy showing reduced, fragmented elastic fibers within the dermis (black arrow). (B) Fragmented elastic fibers highlighted by elastin-specific Elastic Von Gieson (EVG) staining (blue arrow)

Histopathology of a skin biopsy showed reduced, fragmented elastic fibers within the dermis (Figure 5A), fragmented elastic fibers highlighted by elastin-specific Elastic Von Gieson (EVG) staining (Figure 5B), findings consistent with cutis laxa.

In ADCL, cutaneous manifestations often appear later in life, similar to the pattern observed in this patient. Visceral involvement is usually absent or mild.

The patient was counselled regarding the diagnosis and its potential long-term implications and was advised to maintain regular follow-up for monitoring of future complications. Sun-protection was advised. He was also referred to a psychiatrist for the management of major depressive disorder. Considering the extensive cutaneous involvement and the financial restraint, the patient and his family members opted against any reconstructive and cosmetic surgery.

## Discussion

Cutis laxa represents a heterogeneous group of inherited and acquired disorders affecting elastic fiber assembly and integrity [6]. The inherited forms include autosomal dominant, autosomal recessive, and X-linked variants, among which the autosomal dominant subtype generally follows a milder course with near-normal life expectancy. Clinical onset in inherited cutis laxa may vary widely, occurring at birth or emerging later in childhood or adolescence, as seen in our patient [7].

ADCL is characterized by significant clinical variability and comparatively limited systemic involvement. Mutations in the *ELN *gene, which encodes elastin, are the most frequently implicated genetic abnormality in ADCL [8]. While *ELN *mutations are also associated with supravalvular aortic stenosis (SVAS), the two disorders arise from distinct pathogenic mechanisms [8]. ADCL is typically linked to mutations in exons 30-34 of *ELN*, resulting in dominant-negative elastin proteins that disrupt normal fiber assembly and lead to progressive skin laxity [8]. Additional genes reported in ADCL pathogenesis include *FBLN5 *(*fibulin-5*) and *ALDH18A1 *[6].

Systemic manifestations in ADCL are usually mild. Pulmonary involvement, when present, may include emphysema, bronchiectasis, or small-airway disease [6]. Gastrointestinal and genitourinary abnormalities, such as hernias and diverticula, may occur and were noted in our patient. In contrast to autosomal recessive cutis laxa (ARCL), cardiac involvement is uncommon in ADCL. Joint mobility is typically normal, without hyperextensibility [2]. The absence of a family history in our case suggests the possibility of a de novo mutation, which is well recognized in ADCL.

There is no curative therapy for cutis laxa, and management remains supportive and multidisciplinary. Addressing cosmetic concerns, providing psychological and psychiatric support, and guiding patients through the psychosocial impact of the disease are essential components of care. Long-term follow-up is recommended to monitor for potential visceral complications [9]. Reconstructive or cosmetic procedures may be offered when indicated, with counselling regarding the likelihood of recurrence and realistic expectations of surgical outcomes. Although visceral involvement is generally limited, periodic cardiovascular and pulmonary evaluations are advisable. Genetic counselling is also important to inform patients about inheritance patterns and the risk of transmission to future offspring [6]. Additionally, emphasis on strict photoprotection is warranted, as ultraviolet radiation can accelerate elastin degradation and exacerbate disease progression [9].

## Conclusions

ADCL is an uncommon connective tissue disorder with significant clinical variability and generally mild systemic involvement. Early recognition is crucial to prevent potential complications, provide appropriate genetic counselling, and support patients in managing the psychosocial impact of the disease. To the best of our knowledge, this represents one of the first reported cases of ADCL from Pakistan, contributing valuable insight to the literature from our population.
